# Coregulation of the Na/K pump and the h-current as a mechanism for robust neuromodulation

**DOI:** 10.1186/1471-2202-16-S1-P42

**Published:** 2015-12-18

**Authors:** William Barnett, Daniel Kueh, Ronald Calabrese, Gennady Cymbalyuk

**Affiliations:** 1 Neuroscience Institute, Georgia State University, Atlanta, GA 30303, USA; 2 Department of Biology, Emory University, Atlanta, GA 30322, USA

## 

To achieve the behavioral flexibility necessary for survival in a variable environment, neuronal circuits have to produce activity over a broad range of functional characteristics. Central pattern generators (CPGs), rhythmically active neuronal networks that control motor functions such as breathing, swimming, and walking, are ideal systems for addressing questions about how neuromodulation efficiently adjusts network output to meet environmental demands. Neuromodulators modify the dynamics of CPGs by orchestrating changes in multiple ionic channels and the electrogenic pump. The Na+/K+ pump has been implicated as critical in the dynamics of CPGs [1,2]. In the leech heartbeat CPG, the Na+/K+ pump is a target for neuromodulation. The neuropeptide myomodulin decreases the burst period; it increases the h-current and decreases the Na+/K+ pump current.

We developed a model of the leech heart interneuron (HN) including the Na+/K+ pump current and intracellular Na+ dynamics. We separately considered the HN and pairs of HNs coupled through mutual inhibition that form half-center oscillators (HCOs). HCOs form the kernel of the leech heartbeat CPG. To investigate the role of the h-current and the Na+/K+ pump current, we changed corresponding biophysical parameters of these currents. We investigated eight model instantiations representing combinations of three experimental treatments: the blockade of chemical synapses representing application of bicuculline, the blockade of h-current representing application of Cs+, and the enhancement of the h-current and inhibition of the Na+/K+ pump current representing the application of myomodulin.

Experiments with Cs+ and myomodulin showed the separate effects of myomodulin on the h-current and the Na+/K+ pump current [1]. Experimental application of myomodulin decreases the period of oscillatory activity by 17%. Application of Cs+ increases the period of bursting by 24% relative to control. The application of myomodulin in addition to Cs+ decreases the period of bursting by 12% relative to treatment with Cs+. The model captures the qualitative trends in change of cycle period observed in experiments with myomodulin and Cs+. Since the application of myomodulin decreases the period of activity when the h-current is present or absent, the Na+/K+ pump current plays a significant role in the dynamics of the HCO.

We investigated activity of the model with different values of the maximal conductance of the h-current, ${\bar{\text{g}}}_{\text{h}}$ and the maximal pump current, ${\text{I}}_{\text{Pump}}^{\text{Max}}$, for isolated neurons and HCOs. We found ranges of parameters where neurons showed bursting activity. In the HCO, we identified the region of the parameter space that supported functional bursting activity. Joint changes of ${\text{I}}_{\text{Pump}}^{\text{Max}}$ and ${\bar{\text{g}}}_{\text{h}}$ allows neurons to preserve functional activity over a larger span of ${\text{I}}_{\text{Pump}}^{\text{Max}}$ and ${\bar{\text{g}}}_{\text{h}}$ and to produce functional activity with a larger range of period.
